# Psychometric Properties of a German Online Version of the Gudjonsson Suggestibility Scale 1

**DOI:** 10.3389/fpsyg.2021.718805

**Published:** 2021-10-01

**Authors:** Fee-Elisabeth Hein, Vera Scheuble, André Beauducel, Anja Leue

**Affiliations:** ^1^ Institute of Psychology, University of Kiel, Kiel, Germany; ^2^ Institute of Psychology, University of Bonn, Bonn, Germany

**Keywords:** suggestibility, online GSS 1, factorial validity, measurement invariance, norms

## Abstract

Suggestibility is a trait dimension that has been differentiated into Yield and Shift dimensions. Yield refers to the susceptibility to suggestive item content in a first question series (Yield 1) and a second question series following negative feedback (Yield 2). Shift describes the tendency to change answers over the two series of questions depending on social pressure. This study aimed at investigating the psychometric properties and the factor structure of a German online version of the Gudjonsson Suggestibility Scale 1 (GSS 1) and measurement invariance of suggestibility scores for gender and research institution. A total of *N*=560 (*n*=287 female; age: *M*=24.20, *SD*=4.60years) students participated in the study. We present Stanine norms for the application of the online GSS 1. Results supported the theoretical basis of the GSS by revealing the two expected suggestibility factors: Yield and Shift. As expected, a leading factor and a non-leading factor were identified for Yield 1 and Yield 2 and a single factor for Shift. We report psychometric properties (e.g., item difficulty, part-whole corrected item-total correlations, reliability coefficients). We compare the factorial structure of the German online GSS 1 with former versions of the GSS 1. Our data suggest widely measurement invariance for gender and research institution on Yield 1 and Yield 2.

## Introduction

Interrogative suggestibility is defined as “[…] the extent to which, within a closed social interaction, people come to accept messages communicated during formal questioning, as the result of which their subsequent behavioral response is affected” (Gudjonsson and Clark, 1986, p. 84). A widely used tool to measure interrogative suggestibility are the Gudjonsson Suggestibility Scales (GSS; Gudjonsson, 1997).

The GSS including GSS 1 and GSS 2 have an identical structure and follow the same administration procedure (Gudjonsson, 1997, p. 4). Both GSS versions differ in the story that is read aloud at the beginning of a standard experimenter-to-participant examination and the questions asked. The story of the GSS 1 is of forensic relevance and tells the story of a woman who is attacked during her holiday in Spain and robbed of her handbag. The story of the GSS 2 is about a couple saving their neighbor’s boy from an accident with his bicycle.

After the story is presented, participants are asked to reproduce all the content they remember from the story (immediate and delayed recall). Furthermore, the GSS contain 20 questions about each story, which can be asked directly after the immediate free recall or with a delay of 50min at the delayed recall stage. All 20 questions are asked twice. Between the first and second round of questions and regardless of the actual answer performance in the first round of questions, participants are given feedback that they have made a number of mistakes and the questions have to be asked again. Of the 20 questions, 15 questions have a leading content, while the other five questions are non-leading. Among the 15 leading questions, five items have a false alternative structure, which means none of the response options fit the closed question (see below). The remaining 10 items have an affirmative structure that tempts the participants to agree with the wrong content of the questions. The scales were developed for forensic, clinical, and research purposes (e.g., Gudjonsson, 2018).

The interview-based original and standard versions of the GSS have been translated from English into several languages including Icelandic (Haraldsson, 1985), Dutch (Smeets et al., 2009), Polish (Polczyk, 2005), Portuguese (Pires et al., 2013), and German (vom Schemm et al., 2006; Gubi-Kelm and Schmidt, 2018) versions of the GSS 1 and Polish (Polczyk, 2005), Japanese (Watanabe et al., 2013), Portuguese (Pires et al., 2013), Italian (Vagni et al., 2015), and German (Wolfradt and Kugener, 1998) versions of the GSS 2. The translated versions have been typically tested in a participant-experimenter interaction in samples smaller than 210 participants. Subsequently, we aim at highlighting (1) evidence of the factorial validity of the GSS 1 and at describing (2) differences of interviewer vs. online GSS 1 opening new insight into individual differences of suggestibility when an online GSS version is applied.

### Structure of the GSS

The GSS differentiate between three aspects of suggestibility (Gudjonsson, 1997). *Yield 1* reflects the acceptance of misinformation suggested by leading questions in a first question series. *Yield 2* reflects the acceptance of the same leading questions in a second question series under the impression of negative feedback provided after the first question series. *Shift* maps the proneness to change one’s original response in either direction (increased suggestibility vs. reduced suggestibility) under the influence of negative feedback or interrogative pressure (Gudjonsson, 1997; Drake, 2014). Subsequently, we exclusively refer to previous studies on the GSS 1. Gudjonsson (1984, 1992) performed a principal component analysis with subsequent Varimax rotation to investigate the factor structure of the GSS 1 (*N*=195). Yield and Shift scores loaded on two different factors. Pearson correlations between Shift and Yield 2 (males: *r*=0.40, *p*<0.01; females: *r*=0.42, *p*<0.01, two-tailed) were about twice as high as between Shift and Yield 1 (males: *r*=0.24, *p*≥0.05; females: *r*=0.15, *p*≥0.05, two-tailed). The GSS 1 showed moderate Cronbach’s alpha coefficients of *α*=0.77 for Yield 1 and *α*=0.67 for Shift (Gudjonsson, 1984). Inter-rater reliability of Yield 1, Yield 2, and Shift ranges from *r*=0.95 to 0.99 in the GSS 1 (Richardson and Smith, 1993).

For the German translation (vom Schemm et al., 2006), 88 persons performed the GSS 1 according to the manual (Gudjonsson, 1997). Results showed somewhat lower means and standard deviations of the subscales and total suggestibility compared to other GSS 1 versions (Gudjonsson, 1997; Polczyk, 2005; Reutemann, 2006). As a confirmatory factor analysis for the German GSS 1 has not yet been performed, we used this method to investigate the factorial validity.

In a German translation of the GSS 1 (Gubi-Kelm and Schmidt, 2019), women achieved higher immediate recall rates for the details of the GSS story than men. Other GSS 1 studies did not reveal evidence of any significantly different suggestibility scores across gender (Gudjonsson, 1997, 2003, p. 379; Gorassini et al., 2006; Pires et al., 2013). Most previous GSS studies investigated student samples (Gorassini et al., 2006; Reutemann, 2006; vom Schemm et al., 2006; Gubi-Kelm and Schmidt, 2018).

### GSS 1: Interviewer vs. Online Versions

An online version of a test has several advantages. Many people from different places can complete the test concurrently. Hence, data from people with a more diverse cultural background can be collected more economically. Furthermore, no participant-examiner-interaction is necessary and each participant gets the same instructions contributing to the objectivity of a psychometric inventory. Yet, before the online version of a test can be applied, it is necessary that test equivalence with its standard form is empirically demonstrated (American Educational Research Association et al., 2014). A psychometric investigation of the adaption of the online version is important because an adapted online GSS requires several format changes compared to the face-to-face version. For example, instructions that an interviewer normally reads aloud are now presented in written format and participants read them by themselves. Moreover, the interrogative impression of the error feedback between the first and the second block of questions during a face-to-face GSS assessment might have a less intense social impact in the online GSS assessment.

In the same line, answers to suggestible questions usually evoke an implicit or explicit feedback from the interviewer that influences the cognitive set of the interviewee for subsequent questions. Positive feedback generally reinforces an applied response strategy. Expected negative feedback can motivate respondents who are confident in their response strategy to invest more effort to generate correct answers from memory (Gudjonsson and Clark, 1986). However, depending on personality traits like anxiety, even small differences in the interviewer’s behavior (e.g., while providing feedback) can affect the extent of the interviewee’s interrogative suggestibility (cf. Baxter and Boon, 2000). Thus, a standardized test procedure could be an option to exclude non-verbal forms of suggestible feedback. One approach toward this direction is to perform computer-aided testing. The GSS 1 have already been adapted into an English online version (Gorassini et al., 2006) and the GSS 2 into a Japanese online version (Wachi et al., 2019). Both online versions compared their online data with the original English version of the British normative sample (Gudjonsson, 1997). Although both studies revealed increased Shift and lower Yield scores for the online GSS compared to the standard version, both studies conclude that the online GSS is applicable and suitable for practical or research purposes (Gorassini et al., 2006; Wachi et al., 2019).

### Aims and Hypotheses

The present study investigated psychometric properties of the German online GSS 1 and measurement invariance of its scores for gender and research institutions. We explore and compare psychometric properties by means of item mean values, part-whole corrected item-total correlations, and reliability with previous studies on the GSS 1. We present norms for the subscales and a total GSS score of the online GSS 1 which can be administered in an online single-person research or forensic setting (because of the story content) with a self-defined time limit that is free of social interaction with the experimenter. Moreover, we expect a factor structure similar to the original GSS 1 (Gudjonsson, 1997) including Yield 1, Yield 2, and Shift in a large German sample using the online GSS 1 (hypothesis 1). Since the link between the items and the expected factors has clearly been presented in Gudjonsson (1997), the factor structure could be investigated by means of confirmatory factor analysis. We also presume measurement invariance of the online GSS 1 scores for gender and two research institutions (hypothesis 2).

## Materials and Methods

### Participants

A total of *N*=593 students (*n*=300 female; *n*=287 from the University of Kiel, Germany; *n*=306 from the University of Bonn, Germany) participated in the study. Data were collected online between April 2019 and December 2019. Data of 33 participants had to be excluded due to insufficient German language skills, which were assumed when German language was not reported as mother tongue or second language. The final sample consisted of *N*=560 participants (*n*=287 female; *n*=283 from the University of Kiel; *n*=277 from the University of Bonn). Age ranged from 18 to 60years (*M*=24.20years; *SD*=4.60, median: 23years). The students were recruited *via* social media, student councils in various fields of study, university homepages, and mailing lists. They received 10€ for their participation. The study was approved by the ethics committee of the German Society of Psychologists in May 2017.

### Procedure

To investigate their psychometric properties, we administered the German translation by vom Schemm et al. (2006) of the GSS 1 online to the participants using the online survey software EFS Survey of the company Questback.^1^ The German translation of the GSS 1 was performed in accordance with the guidelines of the International Test Commission (2017). As the study aimed at a German adaption of three questionnaires, participants were asked to complete German translations of the Mood and Anxiety Symptom Questionnaire (MASQ^2^), Rumination Scales (LeMoult et al., 2013), and the GSS 1 (Gudjonsson, 1997). We focus on the investigation of the GSS 1 here. All participants gave written informed consent at the beginning of the examination.

### German Online Adaption of the GSS 1

In the current study, the story of the GSS 1 was presented in written format on a computer screen, not as an auditory, interactive version. According to the manual, the 20 GSS 1 questions can be asked after the immediate recall stage (Gudjonsson, 1984, 1997). The online procedure has already been applied in previous studies (Gorassini et al., 2006; Wachi et al., 2019). The 50-min delay interval and delayed recall stage were not tested here (but see Gubi-Kelm and Schmidt, 2018). Participants were instructed to read the story carefully, as they were then asked to reproduce in a written format everything they remember immediately after reading the story. After the free recall, a first block of 20 questions was asked in a written format about the story. According to the manual (Gudjonsson, 1997), the 20 translated questions consisted of 15 leading questions (five false alternative questions, which are closed questions suggesting two incorrect answer options, and 10 affirmative questions that tempt the participants to agree with the suggested question content) and five non-leading questions. After answering the 20 questions in a multiple-choice format, participants were provided with negative feedback in a written format suggesting too many errors regardless of the real performance (similar as in Gorassini et al. (2006), participants got the feedback “You have made a number of errors. It is therefore necessary to go through the questions once more, and this time try to be more accurate”). Subsequently, the 20 questions were repeated in a second block.

Sum scores of three subscales were computed. “Yield” depicts the tendency to give in to leading questions (Gudjonsson, 1997). “Yield” can be subdivided into the subscales “Yield 1” and “Yield 2.” “Shift” displays the number of discrepancies between the first and second round of questions, with all 20 questions being included in the analysis (Singh and Gudjonsson, 1987; Gudjonsson, 1997). The evaluation of Shift was performed according to the manual by Gudjonsson (1997) and the German coding scheme as in Reutemann (2006). Both guidelines corresponded completely. According to the manual, the sum of Yield 1 and Shift formed the overall suggestibility score for the GSS 1 (Gudjonsson, 1997). We have paid an e-course permission in March 2019 at Taylor & Francis company that allowed us to apply the German translations of the GSS items in our online study.

### Statistical Analysis

Psychometric analysis was performed with SPSS version 23.0 (IBM Corp., 2015). To provide evidence of the factorial validity and measurement invariance of the GSS 1, confirmatory factor analyses (CFA) and multiple-group CFAs were conducted using Mplus Version 8.4 (Muthén and Muthén, 1998-2019). Very good model fit refers to a root mean square error of approximation (RMSEA) of about ≤0.06. Hu and Bentler (1999, p. 6) evaluated model fit thresholds for maximum likelihood method in EQS and recommend a comparative fit index (CFI) ≥0.95 for a very good model fit. Beauducel and Wittmann (2005) evaluated model fit thresholds in LISREL and recommend a CFI≥0.90. As we applied Mplus for CFA modeling and WLSMV as a method, both recommended thresholds do not exactly correspond to the models tested in the present study. Therefore, we conceive a CFI between 0.90 and 0.95 as a good model fit. Model fit indices that differ from these cutoff criteria suggest an acceptable or poor model fit. Methodological papers suggest that the terms “measurement equivalence” and “measurement invariance” can be used synonymously (Vandenberg and Lance, 2000, p. 5). Therefore, we use both terms interchangeably here. Confirmatory factor analysis was not applied to test suggestibility as a total GSS 1 scale because the subscales Yield 1 or Yield 2 and Shift are at least partly technically dependent. All factor loadings are reported for the completely standardized solution (STDYX). Parameter estimates are obtained by means of polychoric correlation estimates. Moreover, a robust asymptotic covariance matrix is used to obtain parameter standard errors (Flora and Curran, 2004, p. 470).

Weighted least square mean and variance adjusted (WLSMV) parameter estimation was used as categorical data were modeled. To test for measurement invariance of the factor structure (i.e., of the scales) across gender and research institution (University of Kiel, University of Bonn), we performed a series of multiple-group CFAs (Vandenberg and Lance, 2000). First, we tested for configural invariance, which implies that the items measure the same number of factors with the same freely estimated and fixed zero loadings across gender and research institution, respectively. Second, we investigated metric invariance which implies that the loadings and the intercorrelations of the factors are equal across gender and research institution, respectively. Finally, we investigate scalar invariance which implies that the item thresholds are equal across gender and research institution, respectively (Putnick and Bornstein, 2016; Seib-Pfeifer et al., 2017; Counsell et al., 2020). A separate multiple-group analysis was calculated for each invariance type. The criterion for flagging measurement invariance of scales is a non-significant *χ*^2^_diff_ value (please see Tables 7 and 9). To indicate the latent mean of the measurement invariance factors, we constrained the group of male participants and participants recruited in Kiel to zero. According to Schmitt et al. (2011, p. 417), “fixing the value of one latent mean at zero means that the other mean parameter is equal to the difference in latent means.” In summary, the following design was used for statistical analysis: 3 (Yield 1, Yield 2, Shift)×3(4) (configural invariance, metric invariance, scalar invariance, and for Yield 1 and Yield 2, scalar invariance and mean differences were disentangled)×2 (gender, research institution).

## Results

### Item Parameters, Descriptives, and Stanine Norms

In Table 1, item difficulties and part-whole corrected item-total correlations are presented for each of the Yield items with a value range from 0 to 1 (0=no Yield; 1=Yield) and for Shift items with a value range from 0 to 1 (0=no Shift; 1=Shift). Most items reached a low item difficulty for Yield 1 and Yield 2, indicating an increased rejection of the suggested question content per item, while high item difficulties reflect an increased yielding tendency (i.e., an agreement with question content). That is, the present sample tended to rarely agree with suggested misinformation. Very low item difficulty could be observed among Shift items, indicating that participants rarely changed their answers to any item between the question series. Part-whole corrected item-total correlations were rather small for Yield 1, Yield 2, and Shift (Table 1).

**Table 1:** Item difficulty and part-whole corrected item-total correlation for calculated Yield and Shift Items (values from 0 to 1). The sequence of the numbered items corresponds exactly to the numbered item sequence in the English GSS 1.

	item difficulty			Part-whole corrected item-total correlation		
	Yield 1	Yield 2	Shift	Yield 1	Yield 2	Shift
Item 1	–	–	P_1_ =0.09	–	–	*r*_1_ =0.12
Item 2	P_2_ =0.18	P_2_ =0.21	P_2_ =0.11	*r_2_* =0.13	*r*_2_ =0.26	*r*_2_ =0.29
Item 3	P_3_ =0.00	P_3_ =0.02	P_3_ =0.02	*r*_3_ =0.02	*r*_3_ =0.27	*r*_3_ =0.23
Item 4	P_4_ =0.08	P_4_ =0.16	P_4_ =0.11	*r*_4_ =0.19	*r*_4_ =0.22	*r*_4_ =0.22
Item 5	–	–	P_5_ =0.18	–	–	*r*_5_ =0.17
Item 6	P_6_ =0.02	P_6_ =0.05	P_6_ =0.05	*r*_6_ =0.18	*r*_6_ =0.26	*r*_6_ =0.25
Item 7	P_7_ =0.48	P_7_ =0.52	P_7_ =0.26	*r*_7_ =0.15	*r*_7_ =0.19	*r*_7_ =0.16
Item 8	P_8_ =0.06	P_8_ =0.09	P_8_ =0.08	*r*_8_ =0.11	*r*_8_ =0.34	*r*_8_ =0.30
Item 9	–	–	P_9_ =0.03	–	–	*r*_9_ =0.16
Item 10	P_10_ =0.15	P_10_ =0.23	P_10_ =0.19	*r*_10_ =0.17	*r*_10_ =0.29	*r*_10_ =0.33
Item 11	P_11_ =0.03	P_11_ =0.07	P_11_ =0.06	*r*_11_ =0.28	*r*_11_ =0.32	*r*_11_ =0.28
Item 12	P_12_ =0.03	P_12_ =0.04	P_12_ =0.05	*r*_12_ =0.22	*r*_12_ =0.26	*r*_12_ =0.17
Item 13	–	–	P_13_ =0.07	–	–	*r*_13_ =0.23
Item 14	P_14_ =0.03	P_14_ =0.06	P_14_ =0.07	*r*_14_ =0.19	*r*_14_ =0.33	*r*_14_ =0.30
Item 15	P_15_ =0.16	P_15_ =0.20	P_15_ =0.14	*r*_15_ =0.06	*r*_15_ =0.25	*r*_15_ =0.35
Item 16	P_16_ =0.05	P_16_ =0.09	P_16_ =0.09	*r*_16_ =0.10	*r*_16_ =0.36	*r*_16_ =0.31
Item 17	–	–	P_17_ =0.03	–	–	*r*_17_ =0.21
Item 18	P_18_ =0.09	P_18_ =0.16	P_18_ =0.13	*r*_18_ =0.26	*r*_18_ =0.36	*r*_18_ =0.30
Item 19	P_19_ =0.04	P_19_ =0.07	P_19_ =0.06	*r*_19_ =0.17	*r*_19_ =0.29	*r*_19_ =0.40
Item 20	P_20_ =0.02	P_20_ =0.02	P_20_ =0.02	*r*_20_ =0.22	*r*_20_ =0.34	*r*_20_ =0.26

*In the Yield columns, values were only calculated for the 15 leading items, as their sum forms the Yield factor. “–” indicates non-inclusion of values for the 5 non-leading items in the Yield 1 and Yield 2 factors*.

The psychometric classification of reliability coefficients recommended by George and Mallery (2003) suggests that the internal consistency of the GSS 1 was low (Yield 1: Cronbach’s *α*=0.43; Yield 2: *α*=0.65; Shift: *α*=0.66). The application of the split-half method by dividing the total item set into a first half (items 1 to 10) and a second half (items 11–20) resulted in the following Spearman-Brown corrected split-half coefficients: Yield 1=0.45, Yield 2=0.71, and Shift=0.68 (same item length of both halves). To estimate reliabilities that are much closer to the tested factorial CFA model, we computed the squared factor score determinacy which has been shown to be identical to the reliability of the regression factor score (Beauducel et al., 2016) and which, in the present case, corresponds to Hancock’s *H*, the maximal reliability of scores for the respective dimensions (Hancock and Mueller, 2001). We obtained *H*=0.59 for the factor scores of Yield 1, *H*=0.71 for Yield 2, and *H*=0.71 for Shift, which is except from the value for Yield 1 slightly larger than the recommended minimum reliability level between *H*=0.70 and 0.80 (Hancock and Mueller, 2001, p. 209).

Pearson correlations between Yield 2 and Shift (*r(560)*=0.71, *p*<0.01, two-tailed) were significantly higher than between Yield 1 and Shift (*r(560)*=0.35, *p*<0.01, two-tailed), *z*=9.13, *p*<0.01.^3^ Male and female participants did not differ in Yield 1 scores (*F*(1, 558)=0.51, *p*=0.48, $\eta_{p}^{2}$ <0.01), Yield 2 scores (*F*(1, 558)=1.13, *p*=0.29, $\eta_{p}^{2}$ <0.01), Shift scores (*F*(1, 558)=0.42, *p*=0.52, $\eta_{p}^{2}$ <0.01), or total suggestibility of the GSS 1 (*F*(1, 558)=0.02, *p*=0.90, $\eta_{p}^{2}$ <0.01), but in free recall scores with female participants scoring higher than males (*F*(1, 558)=5.08, *p*=0.03, $\eta_{p}^{2}$ =0.01; Table 2). Participants of the University of Kiel and University of Bonn did not differ in Yield 1 scores (*F*(1, 558)=0.36, *p*=0.55, $\eta_{p}^{2}$ <0.01), Yield 2 scores (*F*(1, 558)=0.54, *p*=0.46, $\eta_{p}^{2}$ <0.01), Shift scores (*F*(1, 558)=1.47, *p*=0.23, $\eta_{p}^{2}$ <0.01), the total suggestibility of the online GSS 1 (*F*(1, 558)=0.37, *p*=0.54, $\eta_{p}^{2}$ <0.01), or free recall scores (*F*(1, 558)=0.40, *p*=0.53, $\eta_{p}^{2}$ <0.01; Table 2).

**Table 2:** Means and standard deviations for sum scores of Yield 1, Yield 2, Shift, total suggestibility, and free recall of the GSS 1.

	Male participants (*n*=273)	Female participants (*n*=287)	University of Kiel (*n*=283)	University of Bonn (*n*=277)	Total sample (n=560)
Yield 1	1.40 (1.41)	1.48 (1.31)	1.46 (1.38)	1.42 (1.34)	1.44 (1.36)
Yield 2	1.90 (2.07)	2.07 (1.84)	1.92 (1.95)	2.06 (1.96)	1.99 (1.95)
Shift	1.87 (2.16)	1.76 (1.95)	1.71 (2.14)	1.92 (1.96)	1.81 (2.06)
Total suggestibility	3.27 (3.01)	3.24 (2.64)	3.17 (2.95)	3.34 (2.70)	3.25 (2.83)
Sum score of free recall	19.04 (6.94)	20.31 (6.35)	19.51 (6.71)	19.87 (6.63)	19.69 (6.67)

*The story contains 40 details. By administration, the minimum score of the free recall is zero and the maximum score of the free recall is 40. The minimum score of the free recall GSS 1 online total score in the present sample was zero (n=1) with a reading time of the story of eight seconds. The maximum score of the free recall GSS 1 online total score in the present sample was 37 (n=1) with a reading time of 44s*.

To overcome criticism of possible contamination effects of memory on suggestibility due to participants who possibly wrote down the story, we refer to the maximum score of details for the free recall of the online GSS 1 to the following descriptive parameters: The total score for GSS 1 free recall had a mean of *M*=19.69 (Table 2), a median of 19.5, and a 75 percentile of 24 (i.e., *n*=428 of the *N*=560 participants had a free recall total GSS 1 online score of no larger than 24).

Compared to other studies, the present sample had very low mean values for Yield 1, Yield 2, Shift, and total suggestibility (Table 3). The free recall score also deviates from those found in other studies, but is in comparison in the middle value range (Table 3).

**Table 3:** Comparison of means and standard deviations of the suggestibility scores measured by GSS in different studies.

Study	Type of survey	Sample	N	Yield 1	Yield 2	Shift	Total Suggestibility	Free recall
Present study	German online GSS 1	students	560	1.4	2.0	1.8	3.3	19.7
				(1.4)	(2.0)	(2.1)	(2.8)	(6.7)
Gorassini et al. (2006)	English online GSS 1	students	41	1.7	2.5	4.3	6.0	19.3
				(1.4)	(2.0)	(2.4)^***^	(3.2)^***^	(5.3)
Gubi-Kelm and Schmidt (2018)	German face-to-face GSS 1	students	88	4.8	5.9	4.4	9.2	–
				(2.5)^***^	(3.0)^***^	(2.8)^***^	(4.6)^***^	
Gudjonsson (1997)	English face-to-face GSS 1	general population	157	4.6	5.6	2.9	7.5	21.3
				(3.0)^***^	(3.8)^***^	(2.5)^***^	(4.6)^***^	(7.1)^**^
Reutemann (2006)	German face-to-face GSS 1	students	101	5.5	6.3	3.6	9.1	24.4
				(2.6)^***^	(3.4)^***^	(2.3)^***^	(3.9)^***^	(5.0)^***^
vom Schemm et al. (2006)	German face-to-face GSS 1	students	88	3.9	4.7	2.6	6.5	–
				(2.6)^***^	(3.1)^***^	(2.5)^***^	(4.1)^***^	
Wachi et al. (2019)	Japanese online GSS 2	general population	442	3.2	6.6	5.3	8.5	14.5
				(3.4)^***^	(4.5)^***^	(4.1)^***^	(6.2)^***^	(7.7)^***^

The asterisks indicate significant mean differences between the respective study and the current study. ^**^p (one-tailed)<0.01. ^***^p (one-tailed)<0.001. “–” These studies did not report data of the free recall.

We computed Stanine norms (*M*=5, *SD*=2) for all GSS 1 sum scales (Table 4) and the respective factor scores (Table 5). The factor scores can be computed from the coefficients in Table 6. Since the scores were not normally distributed, we performed normalization according to McCall (1939). This allows users of the German online GSS 1 to transform raw scores into Stanine norms for scales and factor scores and to interpret the Stanine norms of the Yield 1, Yield 2, and Shift scales for single cases.

**Table 4:** Raw scores and stanine norm for the Yield 1 scale, the Yield 2 scale, the Shift scale, and the scale score G (*N*=560).

Yield 1		Yield 2		Shift		G	
Raw score	Stanine	Raw score	Stanine	Raw score	Stanine	Raw score	Stanine
0	1^*^–3	0	1^*^–3	0	3	0	1^*^–2
1	4^*^–5	1	4	1	4^*^–5	1	3–4^*^
2	6	2–3	5^*^–6	2	6	2–3	5
3	7	4	7	3–4	7	4	6
4	8	5–6	8	5–6	8	5–6	7
5–6, 7^*^, 8, 9, 10^*^–11, 12^*^–15^*^	9	7–1213*–15*	9	7–1213*-15*	9	7–9	8
						10–14, 15^*^, 16^*^, 17–18	9

Raw scores of Yield 1, Yield 2, and Shift scales were performed using sum function. Because item 3 had no sufficient variance it was not included in the computation of the sum scale of Yield 1. ^*^Raw scores or Stanine scores are extrapolated because scores did not originally occur in the norm sample (N=560). We recommend to use the upper value of the respective Stanine score interval.

**Table 5:** Factor scores norm for Yield 1, Yield 2, Shift, and G (*N*=560).

Yield 1		Yield 2		Shift		G	
Factor score	Stanine	Factor score	Stanine	Factor score	Stanine	Factor score	Stanine
≤−0.44	1^*^−3	≤−0.59	1^*^−3	≤−0.67	1^*^−3	≤−0.44	1^*^−3
>−0.44	4	>−0.59	4	>−0.67	4	>−0.44	4
						≤−0.32	
				≤−0.58			
≤−0.37		≤−0.44					
>−0.37	5	>−0.44	5	>−0.58	5	>−0.32	5
						≤0.03	
				≤−0.19			
		≤−0.25					
≤−0.31							
>−0.31	6	>−0.25	6	>−0.19	6	>0.03	6
						≤0.50	
				≤0.33			
		≤0.16					
≤0.00							
>0.00	7	>0.16	7	>0.33	7	> 0.50	7
						≤1.00	
				≤0.99			
		≤0.93					
≤0.51							
>0.51	8	>0.93	8	>0.99	8	>1.00	8
						≤1.62	
				≤2.25			
		≤2.03					
≤2.15							
>2.15	9	>2.03	9	>2.25	9	>1.62	9

Shift factor scores were performed using regression factor scores. ^*^Stanine scores 1–2 cannot be computed because these scores did not occur in the norm sample (N=560). Therefore, we recommend to use 3 as the minimal Stanine score.

**Table 6:** B-weights (regression coefficients) and constant for the computation of factor scores.

Yield 1		Yield 2		Shift		G	
Item	B	Item	B	Item	B	Scale^*^	B
02	0.10	02	0.16	01	0.14	Yield 1	0.47
03	0.00	03	2.07	02	0.33	Shift	0.13
04	0.27	04	0.19	03	1.57	Constant	−0.90
06	1.03	06	0.54	04	0.29		
07	0.07	07	0.07	05	0.12		
08	0.44	08	0.64	06	0.71		
10	0.14	10	0.16	07	0.09		
11	2.43	11	0.58	08	0.48		
12	1.17	12	0.95	09	0.70		
14	2.03	14	0.75	10	0.26		
15	0.04	15	0.15	11	0.61		
16	0.30	16	0.51	12	0.46		
18	0.45	18	0.31	13	0.35		
19	0.64	19	0.44	14	0.57		
20	2.61	20	2.44	15	0.37		
Constant	−0.44	Constant	−0.59	16	0.52		
				17	0.88		
				18	0.33		
				19	0.96		
				20	1.48		
				Constant	−0.67		

The binary items (coded as “0” and “1”) are multiplied with their B-weight and aggregated. The constant is added to the respective sum. The items in the Yield 1 column contain the responses to the respective GSS items in the first measurement occasion. Because item 3 had no sufficient variance, it has a zero-weight for the computation of the factor score of Yield 1. The items in the Yield 2 column contain the responses to the respective GSS items in the second measurement occasion. For the coding of Shift, items refer to method section. ^*^The sum of the raw scores for Yield 1 and Shift is used for the G factor score.

### Yield and Shift: Factor Structure of the GSS 1

**Figure 1:**
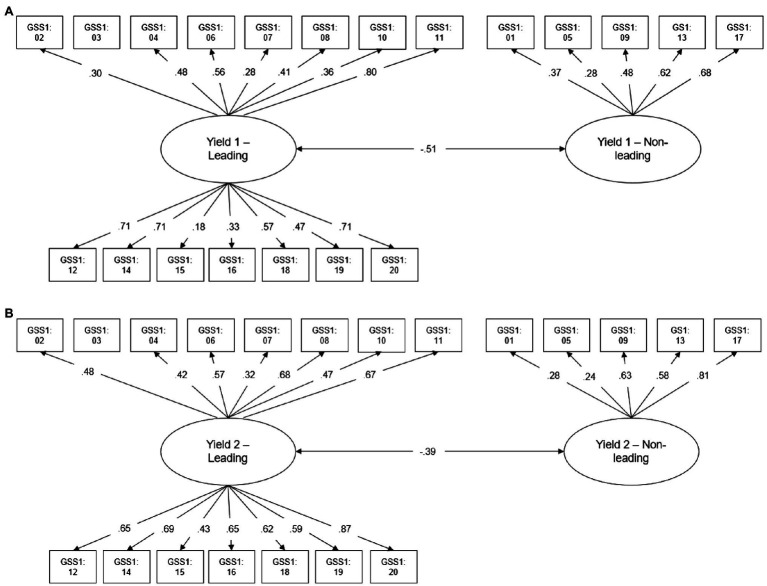
Measurement model (completely standardized solution) for Yield 1 items **(A)** and Yield 2 items **(B)** of a German online GSS 1.

**Figure 2:**
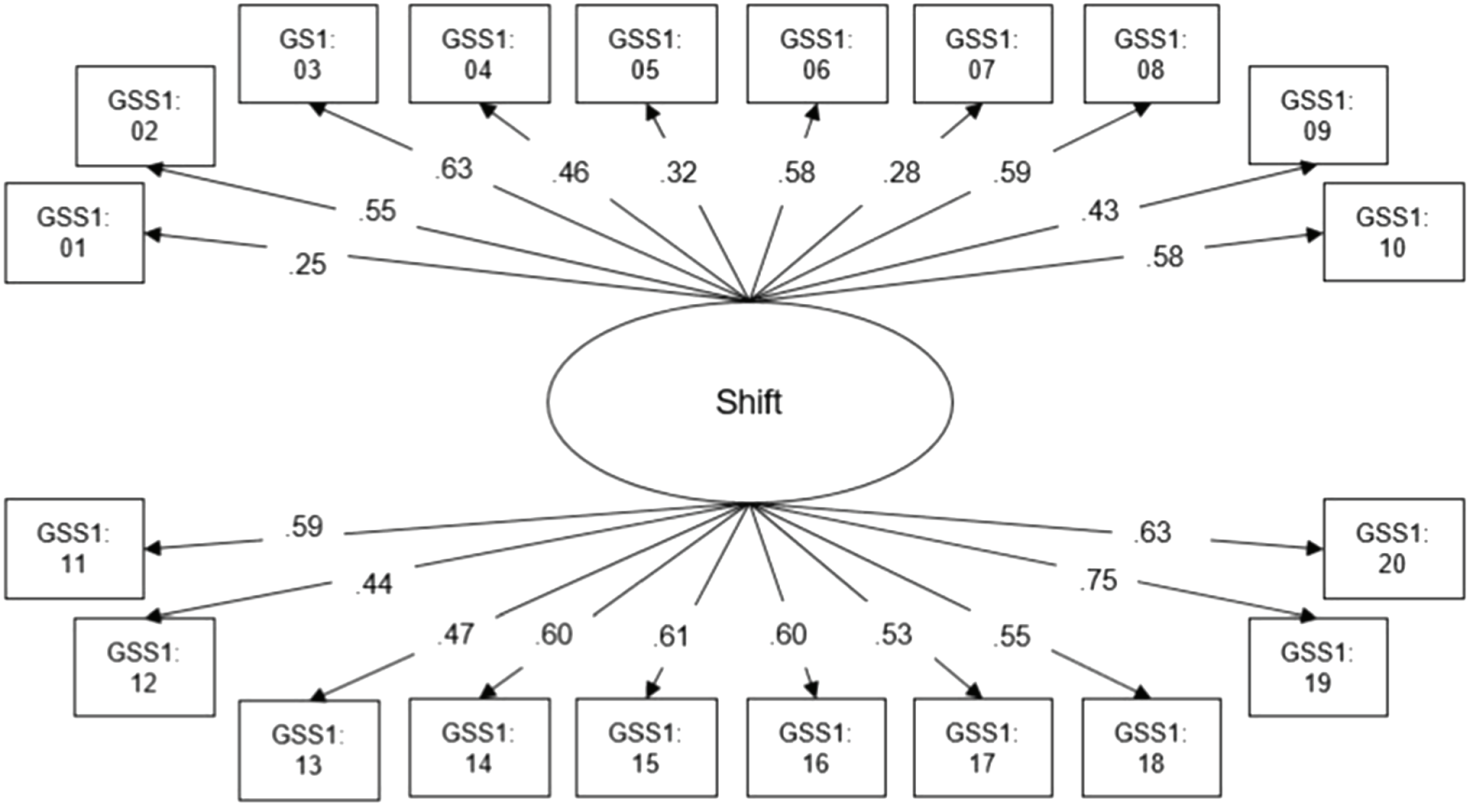
Measurement model (completely standardized solution) for 20 Shift items of a German online GSS 1.

The hypothesized confirmatory two-factor model for Yield 1 had an acceptable model fit (*χ*^2^=174.43, *df*=151, *p=* 0.09; RMSEA=0.02; CFI=0.89). The 20 items loaded on two factors, a Yield 1 leading factor and a Yield 1 non-leading factor (Figure 1A). As item 3 had no variance, Yield 1 and Yield 2 were estimated based on 19 items (14 leading and 5 non-leading items). Secondary factor loadings (below 0.30) were found for items 5, 7, and 15 (Figure 1A). As the acceptance of misinformation suggested by leading questions forms the Yield 1 score, the leading factor is equivalent to the Yield factor. There was a small negative correlation of the leading factor with the non-leading factor indicating that higher values on the leading factor go along with lower values on the non-leading factor (Figure 1A). The hypothesized two-factor model for Yield 2 fitted well with the current data (Yield 2: *χ*^2^=184.02, *df*=149, *p*<0.05; RMSEA=0.02; CFI=0.94). The 19 items loaded on two factors, namely, a Yield 2 leading factor and a Yield 2 non-leading factor (Figure 1B). The items 1 and 5 showed secondary loadings (below 0.30) on the Yield 2 non-leading factor (Figure 1B). A small negative correlation occurred between Yield 2 leading factor and non-leading factor (Figure 1B). Again, since the acceptance of misinformation suggested by leading questions forms the Yield 2 score, the leading factor is equivalent to the Yield factor. The hypothesized single-factor model for Shift showed a very good model fit (*χ*^2^=181.74, *df*=170, *p=* 0.26; RMSEA=0.01, CFI=0.98, Figure 2). Secondary factor loadings below 0.30 were found for items 1 and 7 (Figure 2).

### Yield 1 and 2: Measurement Invariance for Gender

The model fit of a two-factor model for Yield 1 with configural invariance across gender was poor (Table 7). In the model for metric invariance, the loadings and factor intercorrelations were fixed to be equal across groups. The *χ*^2^ difference between the configural invariance model and the metric invariance model and the *χ*^2^ difference between the metric invariance model and the scalar invariance model were not significant (Table 7). As the sequence of model testing indicates measurement invariance across gender, the means of the leading factor and of the non-leading factor of Yield 1 can be compared for males and females. Therefore, mean factor scores of the females were tested against the fixed mean factor scores of the males (Table 8), that is, the mean factor score of males was set to zero (cf. Chen et al., 2019). The differences of mean factor scores equal the standardized mean difference indicated as *d* (Schmitt et al., 2011). The standardized mean difference in the leading factor was not significant, whereas females tended to have slightly larger means on the non-leading factor than males (Table 8).

**Table 7:** Fit of models for the investigation of measurement invariance for gender.

Item set	Type of invariance	*χ*^2^ *(df)*	*χ*^2^ *_diff_ (df_diff_)*	RMSEA	CFI
Yield 1	Configural invariance	333.08 (302), *p* =0.11	–	0.02	0.85
	Metric invariance	341.42 (322), *p* =0.22	17.00 (20), *p=* 0.65	0.02	0.91
	Scalar invariance	364.13 (341), *p* =0.19	26.70 (19), *p* =0.11	0.02	0.89
	Scalar invariance and mean differences	364.13 (341), *p* =0.19	2.38 (2), *p* =.30^a^	0.02	0.89
Yield 2	Configural invariance	352.46 (303), *p* <0.05	–	0.02	0.91
	Metric invariance	376.67 (322), *p* <0.05	28.34 (19), *p* =0.08	0.03	0.90
	Scalar invariance	400.36 (341), *p* <0.05	27.20 (19), *p* =0.10	0.03	0.89
	Scalar invariance and mean differences	400.36 (341), *p* <0.05	0.93 (2), *p* =0.63	0.03	0.89
Shift	Configural invariance	388.77 (340), *p* <0.05	–	0.02	0.92
	Metric invariance	417.78 (360), *p* <0.05	32.43 (20), *p* <0.05	0.02	0.91
	Scalar invariance	439.04 (380), *p* <0.05	22.24 (20), *p* =0.33	0.02	0.90

a The scalar invariance and means differences model has more free parameters than the scalar invariance model, in which the means were fixed. Accordingly, the *χ*^2^ difference test indicates whether free estimation of the group factor means yields a significant fit improvement.

**Table 8:** Latent means of males and females for the latent GSS 1 factors and the standardized mean difference *d.*

	Males *M* (*SE*)	Females *M* (*SE*)	Standardized mean differences (*d*)
Yield 1 – leading	0.00 (0.00)	−0.01 (0.14)	*d* =−0.01, *p* =0.94
Yield 1 – non-leading	0.00 (0.00)	0.24 (0.14)	*d* =0.24, *p* =0.08
Yield 2 – leading	0.00 (0.00)	0.07 (0.12)	*d* =0.07, *p* =0.54
Yield 2 – non-leading	0.00 (0.00)	0.11 (0.14)	*d* =0.11, *p* =0.45
Shift	0.00 (0.00)	−0.07 (0.12)	*d* =−0.07, *p* =0.52

The mean factor scores of males were fixed to zero.

Configural invariance across gender was also investigated for a two-factor model of the Yield 2 items. This model also fitted poor to the data, and the *χ^2^* difference between the configural invariance model and the metric invariance model was marginally significant (Table 7). Although measurement invariance was not perfect (e.g., *χ*^2^ difference between the configural invariance model and the metric invariance model: *p*<0.08; Table 7), the mean differences between females and males were tested and found to be non-significant (Table 8).

### Yield 1 and 2: Measurement Invariance for Research Institution

The two-factor model for Yield 1 with configural invariance across research institution had a poor fit at least for the CFI, not for the RMSEA (Table 9). The loadings and factor intercorrelations were fixed to be equal across groups in the model for metric invariance. The *χ*^2^ difference between the configural invariance model and the metric invariance model was not significant suggesting measurement invariance for Yield 1. Moreover, the *χ*^2^ difference between the metric invariance model and the scalar invariance model was not significant also confirming evidence of measurement invariance (Table 9). Although the fit was poor for the CFI of the configural invariance model, overall measurement invariance for research institution was given as the *χ*^2^ difference scores were non-significant (Table 9). The standardized mean differences for research institution were estimated for the two factors of Yield 1 and were not significant (Table 10).

**Table 9:** Fit of models for the investigation of measurement invariance for research institution.

Item set	Type of invariance	*χ*^2^ *(df)*	*χ*^2^ *_diff_ (df_diff_)*	RMSEA	CFI
Yield 1	Configural invariance	350.35 (302), *p* =0.03	–	0.02	0.79
	Metric invariance	360.83 (321), *p* =0.06	19.33 (19), *p=* 0.44	0.02	0.83
	Scalar invariance	381.51 (340), *p* =0.06	21.90 (19), *p* =0.29	0.02	0.82
Yield 2	Configural invariance	373.10 (338), *p* =0.09	–	0.02	0.94
	Metric invariance	386.18 (358), *p* =0.15	19.89 (20), *p* =0.46	0.02	0.95
	Scalar invariance	404.67 (378), *p* =0.17	17.28 (20), *p* =0.64	0.02	0.96
	Scalar invariance and mean differences	401.82 (377), *p* =0.18	0.71 (3), *p* =0.87	0.02	0.96
Shift	Configural invariance	399.99 (340), *p* <0.05	–	0.03	0.91
	Metric invariance	458.89 (360), *p* <0.05	55.49 (20), *p* <0.05	0.03	0.84
	Scalar invariance	478.54 (380), *p* <0.05	18.46 (20), *p* =0.56	0.03	0.84

a The scalar invariance and means differences model has more free parameters than the scalar invariance model, in which the means were fixed. Accordingly, the *χ*^2^ difference test indicates whether free estimation of the group factor means yields a significant fit improvement.

**Table 10:** Latent means of Research institution (Kiel compared to Bonn) for the latent GSS 1 factors and the standardized mean difference *d*.

	Kiel *M* (*SE*)	Bonn *M* (*SE*)	Standardized mean differences (*d*)
Yield 1 – leading	0.00 (0.00)	−0.08 (0.14)	*d* =−0.08, *p* =0.59
Yield 1 – non-leading	0.00 (0.00)	0.25 (0.14)	*d* =0.25, *p* =0.08
Yield 2 – leading	0.00 (0.00)	0.06 (0.11)	*d* =0.06, *p* =0.59
Yield 2 – non-leading	0.00 (0.00)	0.06 (0.14)	*d* =0.06, *p* =0.68
Shift	0.00 (0.00)	0.11 (0.11)	*d* =0.11, *p* =0.35

The mean factor scores of Kiel were fixed to zero.

The two-factor model for Yield 2 with configural invariance across research institution fitted well to the data in terms of RMSEA and CFI. Neither the *χ*^2^ difference between the configural invariance model and the metric invariance model nor between the metric invariance model and scalar invariance model were significant (Table 9), suggesting measurement invariance across research institutions. The difference in the mean factor scores between research institutions did not differ significantly for Yield 2 (Table 10).

### Shift: Measurement Invariance for Gender

The configural invariance model across gender fitted the data well in terms of RMSEA and CFI (Table 7). According to the *χ*^2^ difference test, the metric invariance model fitted less to the data indicating that the loading pattern was different across gender. The standardized mean differences (Table 8), however, were not significant for gender differences of the Shift scale. Thus, particularly the metric model indicates that the Shift factor had a rather different meaning for males and females. The latent means recommend that women shifted less frequently their answers than men (Table 8). The fit of the scalar invariance model was not significantly worse than the fit of the metric invariance model (Table 7). However, since there was no metric invariance, the mean differences between females and males on the Shift factor were only reported as descriptive statistics in addition to the *χ*^2^ difference tests (Table 8).

### Shift: Measurement Invariance for Research Institution

The single-factor model for Shift with configural invariance across research institution fitted the data well in terms of RMSEA and CFI (Table 9). The *χ*^2^ difference test indicated that the metric invariance model fitted significantly less to the data and the scalar invariance model and the metric invariance model fitted the data equally well (Table 9). As for gender, the standardized mean differences (Table 10), however, were not significant for research institution of the Shift scale. Due to the lack of metric invariance, mean differences between research institutions were only reported as descriptive statistics in addition to the *χ^2^* difference tests (Table 10).

## Discussion

A German online version of the GSS 1 with immediate (not delayed) recall was administered to a large sample to examine its psychometric characteristics, factor structure, and to present Stanine norms. Furthermore, we investigated factorial validity, configural, metric, and scalar invariance across gender and research institutions by means of multiple-group CFAs. Reliability measures were found to be comparable to face-to-face versions of the GSS 1 (Gudjonsson, 1984; Gubi-Kelm and Schmidt, 2018), but no reliability data were reported in previous online GSS studies (Gorassini et al., 2006; Wachi et al., 2019). When based on factor scores, the reliability of Yield 1 increases but is still questionable, whereas the reliabilities of Yield 2 and Shift are acceptable (George and Mallery, 2003). For the first time, Stanine norms of the GSS 1 online version are presented.

Data of the German online GSS 1 confirmed the theoretical basis of the GSS 1 as the two factors Yield and Shift were confirmed in a CFA. Furthermore, consistent with previous studies, Yield 2 and Shift were highly correlated, whereas Yield 1 and Shift correlated moderately. The Yield 1, Yield 2, Shift, and total suggestibility scores of the GSS 1 in the present study were significantly lower compared to previous studies on the GSS 1 (Gudjonsson, 1997; Reutemann, 2006; vom Schemm et al., 2006; Gubi-Kelm and Schmidt, 2018; Wachi et al., 2019). As Gorassini et al. (2006) found similarly low Yield scores in their English online GSS 1, this discrepancy might be attributed to the online format. In contrast to a face-to-face setting, in which the story is read aloud to the participant once, in the present online format, the story was presented visually as a text without a time limit for reading. Thus, participants could read the story as often as necessary to memorize it prior to item presentation, which may have given the current sample a memory advantage over samples tested with the original version with a predefined duration of 1.5–2s per auditorily presented detail (Gudjonsson, 1997, p.11).

In a face-to-face setting, the subsequent 20 questions are asked orally and the participants can answer in own words whatever comes to their mind. In contrast, the online setting provides concrete answer options and participants can choose between “true,” “wrong,” and “I do not know” to affirmative questions or “alternative 1,” “alternative 2,” “I do not know,” and “neither of them” to false alternative questions. Offering answer categories might affect the suggestive effect of leading questions, since the agreement and rejection of the suggested question content are both visually presented as explicit options. This could raise suspicion about the questions suggestive content and trigger a more conscious consideration of the response. An important issue for future research is therefore to investigate whether predefined response options cause reduced suggestibility effects in GSS online application compared to a free answer format.

Importantly, in the total sample, the measurement models for Yield 1 and Yield 2 suggest two-factor models with the 15 leading items of the GSS 1 loading on the leading factor and the five non-leading items loading on the non-leading factor. This factor structure reflects the theoretical basis of the original GSS obtained in exploratory factor analyses (EFA; Gudjonsson, 1997). The measurement model for Shift obtained based on CFA is also comparable to the original GSS investigated by means of an EFA (Gudjonsson, 1997). A good model fit was obtained for a single-factor model, indicating that the difference scores of Yield 1 items and Yield 2 items represent the Shift factor. Thus, it can be suggested that the factor structure of the online GSS 1 equals the face-to-face version. It should, however, be noted that a direct comparison of statistical parameters obtained in CFA and EFA should be performed with caution because the model specification strongly differs between CFA and EFA. As the GSS consist of two versions (GSS 1 and GSS 2) that share the same scale structure, it should be investigated in a future study whether the results on the German online GSS 1 may also be applicable to a German online GSS 2.

Simple group comparisons revealed no differences between male and female participants with respect to Yield 1, Yield 2, Shift, and total suggestibility scores of the online GSS 1. With a multiple-group CFA, the measurement invariance types could be differentiated more precisely. The Yield 1 items were and the Yield 2 items tended to be measurement equivalent across gender. Therefore, mean differences between females and males on the Yield 1 and Yield 2 factors could be interpreted. Consistent with prior studies (Gudjonsson, 1997, 2003; Gorassini et al., 2006; Pires et al., 2013), the results provide no evidence of a gender effect on Yield 1, Yield 2, or total suggestibility except for the non-leading factor of Yield 1. There was a tendency of females to show higher Yield 1 non-leading values than men, indicating that females tend to agree with the correct content of Yield 1 non-leading items more often than men.

No differences were found between the University of Kiel and the University of Bonn with regard to Yield 1, Yield 2, Shift, and total suggestibility scores using a simple group comparison. In order to differentiate the measurement invariance types more precisely, a multiple-group CFA was calculated here as well. For the Yield 1 items, even the configural invariance model did not fit the data very well at least for the CFI for measurement equivalence of research institution. This suggests that model specifications regarding freely estimated and fixed zero loadings and factor loadings on items appear to differ for measuring Yield 1 in both research institutions. Although the fit does not decrease substantially in the metric and scalar invariance models, the low fit of the configural invariance model in terms of CFI implies that a good model fit was restricted to the RMSEA. Invariance across research institution was given for Yield 1 as the *χ*^2^ difference values were non-significant (Table 9). The Yield 2 items tended to be measurement equivalent across research institution (i.e., University of Kiel and the University of Bonn). This is a particularly important finding, as the resource efficiency and location-independent manner is a major advantage of an online vs. face-to-face format (Nayak and Narayan, 2019). As metric invariance was not given for Shift between female and male participants as well as between research institution, we show that factor loadings and intercorrelations showed differential item functioning. Moreover, the Shift items can be assumed to be differentially salient for females and males as well as for participants of both research institutions (cf. American Educational Research Association et al., 2014). Therefore, the differentiation of emic and etic research approaches should be taken into account in future research (cf. Rolland, 2002).

## Limitations and Future Directions

Since participants completed the online survey at home, there was no direct possibility to check whether cheating (e.g., writing down the story) had occurred. However, taking a look at the free recall scores, in the current sample, only one participant reached the maximum of 37 points out of a possible 40, giving the impression that the likelihood of cheating was rather low (see Results). In future studies, new variants of the online survey (e.g., digital rooms) can be enabled with test persons in order to prevent cheating.

Although the present study provides important findings on the psychometric properties, factor structure, and norms of a German online version of the GSS 1, the delayed recall of the German online GSS should be probed in another study and further administration contexts including the relation of suggestibility and false confessions (Gudjonsson, 2021). Thus, currently, we cannot conclude on similarities between the German online GSS 1 and the (paper-pencil/interview) German face-to-face GSS 1 regarding memory performance and total suggestibility. Moreover, we aim at investigating the external validity in another study by relating GSS 1 data to event-related potentials like error-related negativity. Future research might also address the relationship between response biases and GSS subscales. Currently, the variation of the item difficulty in Table 1 indicates that there is at least no evidence of acquiescence in our data. Deng and Chan (2017) compared Cronbach’s alpha coefficients and McDonald’s omega as reliability coefficients for performance scales. Future research might also compare reliability coefficients not only for items and factor scores (see Results section) but also for Cronbach’s alpha coefficients and McDonald’s omega in personality studies including GSS.

## Conclusion

This study demonstrates the factorial validity and norms of the German online GSS 1 for assessing Yield 1, Yield 2, Shift, and total suggestibility (with immediate recall). Standardized online assessment in an economical manner is important for panel research and when varying social influences on GSS findings should be reduced for the sake of test objectivity (American Educational Research Association et al., 2014).

## Data Availability Statement

The raw data supporting the conclusions of this article will be made available by the authors, without undue reservation.

## Ethics Statement

The studies involving human participants were reviewed and approved by Ethics Committee of the German Society of Psychologists. The patients/participants provided their written informed consent to participate in this study.

## Author Contributions

AB and AL contributed to the study conceptualization and funding acquisition. F-EH and VS collected and prepared data. F-EH, AB, and AL performed statistical analyses. F-EH wrote the first draft of the manuscript. F-EH, VS, AB, and AL wrote sections of the manuscript. All authors contributed to manuscript revision, read, and approved the submitted version.

## Funding

The study was funded by the German Research Foundation to the third and fourth authors (BE 2443/11-1, LE 2240/6-1).

## Conflict of Interest

The authors declare that the research was conducted in the absence of any commercial or financial relationships that could be construed as a potential conflict of interest.

## Publisher’s Note

All claims expressed in this article are solely those of the authors and do not necessarily represent those of their affiliated organizations, or those of the publisher, the editors and the reviewers. Any product that may be evaluated in this article, or claim that may be made by its manufacturer, is not guaranteed or endorsed by the publisher.

## Footnotes

^1^ https://www.unipark.com/

^2^Watson, D., and Clark, L. A. (1991). The Mood and Anxiety Symptom Questionnaire. Unpublished Manuscript, University of Iowa, Department of Psychology, Iowa City.

^3^ https://www.psychometrica.de/korrelation.html
